# Microstructure of Copolymers of Norbornene Based on Assignments of ^13^C NMR Spectra: Evolution of a Methodology

**DOI:** 10.3390/polym10060647

**Published:** 2018-06-09

**Authors:** Laura Boggioni, Simona Losio, Incoronata Tritto

**Affiliations:** Istituto per lo Studio delle Macromolecole (ISMAC), Consiglio Nazionale delle Ricerche (CNR), Via E. Bassini 15, 20133 Milano, Italy; boggioni@ismac.cnr.it (L.B.); s.losio@ismac.cnr.it (S.L.)

**Keywords:** cyclic olefin copolymers, terpolymers, ^13^C NMR, microstructure

## Abstract

An overview of the methodologies to elucidate the microstructure of copolymers of ethylene and cyclic olefins through ^13^C Nuclear magnetic resonance (NMR) analysis is given. ^13^C NMR spectra of these copolymers are quite complex because of the presence of stereogenic carbons in the monomer unit and of the fact that chemical shifts of these copolymers do not obey straightforward additive rules. We illustrate how it is possible to assign ^13^C NMR spectra of cyclic olefin-based copolymers by selecting the proper tools, which include synthesis of copolymers with different comonomer content and by catalysts with different symmetries, the use of one- or two-dimensional NMR techniques. The consideration of conformational characteristics of copolymer chain, as well as the exploitation of all the peak areas of the spectra by accounting for the stoichoimetric requirements of the copolymer chain and the best fitting of a set of linear equation was obtained. The examples presented include the assignments of the complex spectra of poly(ethylene-*co*-norbornene (E-*co*-N), poly(propylene-*co*-norbornene (P-*co*-N) copolymers, poly(ethylene-*co*-4-Me-cyclohexane)s, poly(ethylene-*co*-1-Me-cyclopentane)s, and poly(E-*ter*-N-*ter*-1,4-hexadiene) and the elucidation of their microstructures.

## 1. Introduction

Nuclear magnetic resonance (NMR) spectroscopy today is a powerful tool for the elucidation of polymer structure and dynamics. In particular, solution ^13^C NMR is indispensable for assigning polymer microstructures. Significant advances have been made in NMR instrumentation and sophisticated pulse sequences, which allow us to solve interesting problems of polymer science [1]. However, it is not yet possible to predict NMR resonance frequencies accurately enough to completely characterize polymer microstructures despite the progress made in Quantum Mechanical theory and calculation methods. This is because the magnetic shielding of nuclei of flexible molecules like polymers are influenced by microstructures, and predictions must be averaged over all the conformations permitted to the chain with a specific microstructure, that is, each nucleus (i) experiences a local magnetic field **B_loc(i)_** that depends on the local conformation of the vicinal carbons, which are related to the microstructure of the vicinal polymer segments.

***Microstructure*** → ***conformation*** → **B_loc(i)_** → **δ^13^C_i_** [2]

Empirical nuclear shielding effects [3,4,5,6], produced by substituents α, β, and γ to a carbon atom, were successfully used to understand polymer microstructure from their ^13^C NMR spectra. Unrevealing the conformational origin of the nuclear shielding produced by γ substituents was fundamental, i.e., a γ substituent could only shield a ^13^C nucleus if the central bond between them produced a proximal arrangement by adopting a gauche conformation, that is, when the rotational internal ***C―****C*_(α)_*―*C_(β)_*―*C_(_γ_)_ is gauche, the resonance of **C** is shifted by about 5 ppm upfield with respect to trans [6].

**Scheme 1:**
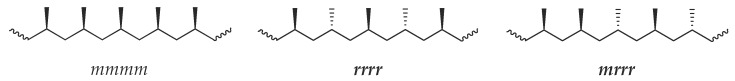
Examples of regioregular polypropylene sequences.

α-, β-, and especially the conformationally sensitive γ-effects were used to assign the NMR spectra and determine the microstructures of polymers in solutions and melts, where they are conformationally flexible [2,6,7,8,9,10,11] .The first applications of the rotational state isomeric model for describing the conformational statistics date back to the late 70s and early 80s in the case of polypropylene (PP) [6,9]. Nowadays, PP resonances in high-resolution solution ^13^C NMR spectra exhibit a sensitivity to stereosequences at the undecad level. This means that eleven repeat unit fragments of PP different only in one diad, which is *meso* (*m*) or *racemic* (*r*) (Scheme 1) may be detected, i.e. a 0.03 ppm difference in the resonance frequencies of the methyl carbons in the central repeat units of mmmmmmmrmr and mmmmmmmrmm PP undecads was observed [12]. More typically ^13^C NMR is usually only sensitive to tetrad and pentad stereosequences in homopolymers and triad comonomer sequences in copolymers. However, whenever a new polymer structure is synthesized, the structural sensitivity of ^13^C NMR allows us to observe a multitude of ^13^C resonances, and it is challenging to assign them to specific microstructures. 

**Scheme 2:**
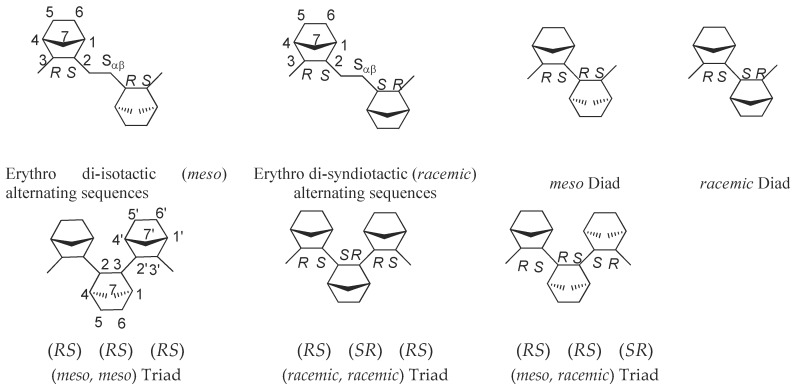
Segments of E-*co*-N copolymer chain with the adopted numbering.

**Scheme 3:**
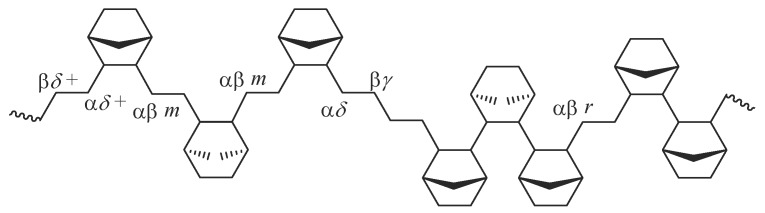
A random E-*co*-N copolymer chain with nomenclature of secondary (S) ethylene carbons (S is omitted for sake of simplicity).

Here, we focus on our efforts to elucidate the microstructure of cyclic olefin-based copolymers (COC), by ^13^C NMR analysis, specifically of ethylene (E)-norbornene (N) copolymers. The spectra of E*-co*-N copolymers are quite complex because the norbornene unit in the polymer chain contains two stereogenic carbons. In addition, the chemical shifts of these copolymers do not follow simple additive rules, due to the bicyclic nature of the norbornene structural units (see Scheme 2 and Scheme 3). 

Scheme 2 illustrates some of the various possible types of chain fragments (alternating and blocks) and also distinguishes between *meso* (*m*) and *racemic* (*r*) alternating units and between *meso* and *racemic* ENNE and ENNNE sequences, where norbornene underwent 2,3 c*i*s-*exo* insertion.

Among COCs with various comonomer contents and microstructures E-*co*-N copolymers are the most versatile and interesting ones, and one of the new families of olefinic copolymers made available by metallocenes. They were first synthesized by Kaminsky with *ansa*-zirconocenes in 1991 [13], and aroused much interest because of their excellent thermal and optical properties [14,15,16]. Owing to their unique combination of properties, they are engineering polymers that are produced and commercialized. They are usually amorphous and display a wide range of glass transition temperatures *T*_g_, from room temperature to about 220 °C, largely determined by chain composition and stereochemistry. 

The thorough microstructure investigation of E-*co*-N copolymers has taken several years [17,18,19,20,21,22,23,24,25,26,27,28,29,30,31,32]. A number of groups dedicated great efforts to assign the ^13^C NMR spectra of E-*co*-N copolymers. Advances in understanding E*-co*-N and P-*co*-N copolymer microstructure through ^13^C NMR signal assignments will be reviewed along with various methodologies utilized to achieve them. Few examples of recent novel assignment of poly(ethylene-*co*-4-Me-cyclohexane)s and poly(ethylene-*co*-1-Me-cyclopentane)s and of even more complex spectra of cyclic olefin terpolymers poly(E-*ter*-N-*ter*-1,4-hexadiene) will be given.

**Figure 1:**
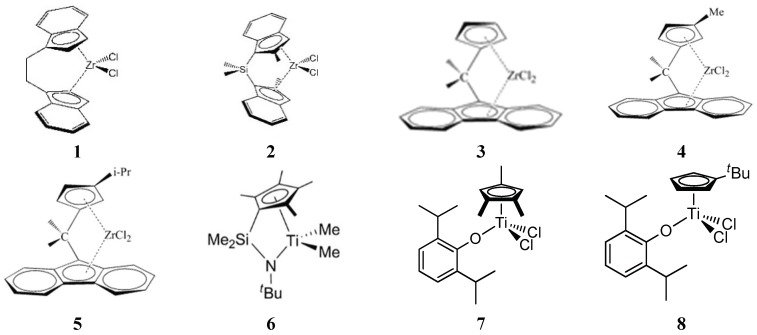
Structures of the metallocenes and half-metallocenes used for copolymerization reported.

In Figure 1 the metallocene and half-titanocene precatalysts, used along with methylaluminoxane (MAO) cocatalyst for the synthesis of copolymers whose microstructure will be elucidated, are displayed. 

## 2. Results and Discussion

### 2.1. Methodologies for Signal Assignments

The methodologies used to make ^13^C NMR signal assignments that allow one to clarify the microstructure of novel cyclic olefin copolymers will be illustrated with the example of E-*co*-N copolymers. 

Assigning ^13^C NMR spectra of norbornene-based copolymers includes synthesis of copolymers with different *comonomer* content (obtained by catalysts with different symmetries), synthesis of copolymers with monomers ^13^C- selectively enriched, synthesis of model compounds, use of mono- or two-dimensional NMR techniques, study of conformational characteristics of copolymer chain, and least-squares fitting of peak areas.

#### 2.1.1. NMR Pulse Sequences

Distortionless enhancement by polarization transfer (DEPT) ^13^C spectra. allows one to distinguish between methyl or methine and methylene carbons; the methine and methyl signals appear positive, while the methylene signals are negative. Thus, DEPT experiments permit a first general assignment of methylene (C7) and methine (C1/C4 and C2/C3) carbon atoms.

#### 2.1.2. Series of Copolymers with Different Comonomer Content

The comparison of spectra of copolymers with different norbornene content, obtained by catalysts with different symmetries, has facilitated the assignment of a number of signals. Unfortunately, this approach when used alone is rather limited in assigning E-*co*-N copolymer spectra [23,24].

#### 2.1.3. ^13^C Enrichment

Comparison between ^13^C NMR spectra of E-*co*-N copolymers of monomers with natural abundance of ^13^C and those obtained with ^13^C_1_-enriched ethylene or ^13^C_5/6_-enriched norbornene allowed Fink to determine the number of C5/C6 or ethylene signals and to make important progress in their assignments [17].

#### 2.1.4. Conformational Characteristics of the Copolymer Chain

Before our studies, no published paper considered the possibility of isotactic or syndiotactic types of regularity for alternating NENEN nor of meso/racemic norbornene diads (ENNE sequences). The elucidation of the conformational structure of the chain of E-*co*-N copolymers by molecular mechanics calculations and the correlation between conformation and ^13^C NMR chemical shifts allowed us to make significant progress [19]. Conformer populations of the stereoregular and stereoirregular polynorbornene and alternating (N-E)x chains and of copolymer chains containing NN, EEE and NNN sequences were computed and allowed us to predict stereochemical shifts. For the first time, it was possible to distinguish between meso and racemic NEN sequences and to assign signals of the two methines C2, Cl of the cyclic unit and of the CH_2_ of ethylene in regularly alternating isotactic and syndiotactic copolymers, and the signals of the two methines of an N unit in a NEE... sequence [19,20].

#### 2.1.5. Model Compounds

In principle, the synthesis of model compounds of a copolymer sequence offers the best approach to making certain assignments of the signals of the sequence and to successfully determine copolymer structure and tacticity. However, the synthesis of model compounds is not always accessible to the synthesis of hydrooligomerization followed by isolation of hydroisomers, which can be model compounds of a segment of the copolymer chain, used to investigate copolymer structure and tacticity. However, the higher the oligomerization number, the higher the number of possible stereisomers and more difficult the separation process. When available, these assignments are very valuable and useful for understanding the shifts. Although in the case of poly-norbornene and of its higher oligomers, due to strong steric interactions between non-adjacent units, which induce large deformations of the torsional angles and of the ring geometry, the results of molecular mechanics show that dimers and trimers are poor models [21]. 

#### 2.1.6. Two-Dimensional NMR Techniques

Two-dimensional NMR techniques, including homonuclear ^1^H-^1^H, ^13^C-^13^C and heteronuclear ^1^H-^13^C experiments, have been helpful in extending signal assignments. 

INADEQUATE ^13^C-^13^C correlated NMR spectra have helped to attribute resonances of NN diads and to correct previous assignments. By using ^13^C-^1^H correlations, HMQC (Hetero Nuclear Multiple Quantum Coherence for one-bond correlations, and HMBC (Heteronuclear Multiple Bond Correlation) for two-or three-bond correlations, Bergstrom et al. identified C5/C6 and C2/C3 signals of norbornene diads [22].

A set of copolymers was extensively investigated by applying the heteronuclear ^1^H-^13^C experiments, namely gHSQC (gradient assisted Heteronuclear Single Quantum Coherence) and ^1^H-^13^C gHMBC experiment (gradient assisted Heteronuclear Multiple Bond Correlation) together with the not reported homonuclear ^1^H-^1^H data.

The gHSQC experiment provides correlations between the resonances of ^1^H and ^13^C atoms having one-bond scalar couplings (^1^*J*_CH_), thus giving information on all the direct one bond proton-carbon correlations. 

Once assigned, all the direct ^1^H-^13^C connections, the ^1^H-^13^C gHMBC experiments allow for a complete and unambiguous resonance assignment. A set of copolymers was extensively investigated by applying gHSQC and gHMBC, allowing us to assign new resonances depending on the comonomer sequences at the triad level.

#### 2.1.7. Ab Initio Theoretical ^13^C NMR Chemical Shifts, Combined with R.I.S. (Rotational-Isomeric State) Statistics

Ab initio computations can be a great tool for the elucidation of complex spectra and for the determination of polymer and copolymer microstructures. A proper model compound and a thorough conformational analysis of the models must be selected and associated with the quantomechanical study. On the basis of previous computations defining the main conformational characteristics of E-*co*-N copolymers, a thorough test of ab initio ^13^C chemical shifts computations [gauge-invariant atomic orbitals (GIAO)] on known cases agreed well with experimental data, especially with the MPW1PW91 density functional theory (DFT), on properly energy-minimized structures. This method nicely confirmed signal assignment of ENNE sequences in spectra of E-*co*-N copolymers, where NN microblocks induce strong effects arising from ring distortions [30].

#### 2.1.8. Set of Equations and Least-Squares Fitting of Peak Areas

A procedure for computing the molar fractions of the stereosequences that describe the E*-co-*N copolymer chain microstructure, has been conceived, which also allowed us by *trial-and-error* to extend the assignment of unknown signals of ^13^C NMR spectra of E*-co-*N copolymers. The analysis of the spectra gives a certain number of peak integrals, each peak c being related to one or more signals. For each peak, it is possible to write a linear equation defining the normalized integral as a function of the unknown molar fractions. The ^13^C NMR signals are associated with carbons of a stereosequence, thus generating a set of equations, whose best-fitting solution determines the microstructure of the copolymer chain and confirms or discards new assignments. This is based on the assumption that the area of a signal is proportional to the population of the carbons generating that signal. Thus, the normalized peak area *NPA*(*i*) of a signal due to carbon *C*_i_ contained *n*_i_ times in the central monomer unit of sequence *S* represents the molar fraction *f*(*C*_i_) of carbon *C*_i._

### 2.2. Microstructural Analysis of E-co-N Copolymers 

The microstructural analysis by ^13^C NMR of E-*co*-N copolymers with different microstructures was completely obtained at triad, tetrad and sometimes at pentad level by the methodology explained above that exploits all the peak areas of the spectra and accounts for the stoichoimetric requirements of the copolymer chain [29,30]. The procedure to analyze the ^13^C NMR spectra of E-*co*-N copolymers is based on proper division of the polymer chain into fragments, in line with the assignment level. The observed peak areas of the greatest possible number of attributed ^13^C NMR signals assignable are employed to generate a set of linear equations where the molar fractions are the variables. In summary, for a given spectrum, it is possible to write a linear equation from each distinctly measured peak area:

$$NPA=normalized\;peak\;area=\;\underset{i}{\sum }c_{i}f_{i}$$

where *c*_i_ = coefficients and *f*_i_ = unknown molar fractions = variables.

Least-squares fitting of the set of equations so obtained gives the best solution for the molar fractions, which define the chain microstructure to achieve new signal assignments. The microstructure of an E-*co*-N copolymer at tetrad or higher level, which distinguished between meso and racemic contributions to alternating and block sequences, was obtained. This allowed us to determine the reactivity ratios, to test the first-order and the second-order Markov statistics and thus to have information on the copolymerization mechanisms [30,31,32,33]. Examples of advancements achieved with this methodology are given below.

#### 2.2.1. Microstructure Alternating E-*co*-N Copolymers with Mid-Low N Content 

**Scheme 4:**
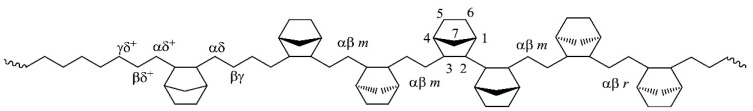
A typical E-*co*-N copolymer chain, containing norbornene in alternating sequences with differences in stereochemistry or norbornene isolated between ethylene blocks.

A typical E-*co*-N copolymer chain, containing norbornene in alternating sequences with differences in stereochemistry or norbornene isolated between ethylene blocks, is sketched in Scheme 4, along with the adopted carbon numbering and denomination. 

**Figure 2:**
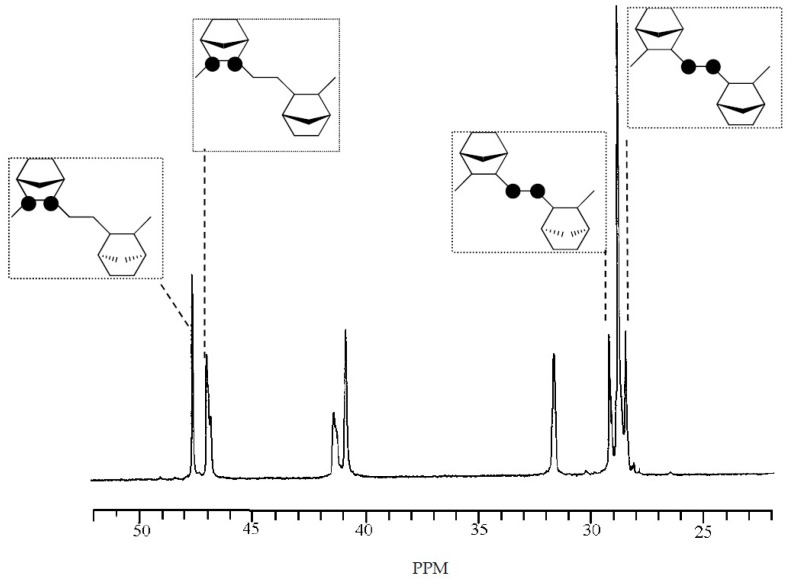
^13^C NMR spectrum of an alternating atactic E-*co*-N copolymer prepared with **6** catalyst. [34].

C_1_-symmetric, bridged metallocenes and monocyclopentadienyl titanium amido complexes (**6** in Figure 1) are able to yield mainly alternating E-*co*-N copolymers. Figure 2. displays the ^13^C NMR spectrum of a E-*co*-N copolymer prepared with **6**/MAO, containing about 43.6 mol % of N, along with the signal assignment. 

Assignments indicated in Figure 2 of two methines C2 of the cyclic unit and of the ethylene CH_2_ signals in regularly alternating isotactic and syndiotactic NEN sequences, as well as of the S_αδ_ signals and the S_βγ_ and S_γδ_ signals shifted lowfield with respect to S_βε_ e and S_δε_, were respectively achieved through comparison of conformer populations [19]. Utilizing the observed peak areas of the assigned ^13^C signals and accounting for the stoichiometric requirements, it was possible to compute the molar fractions of the stereosequences, which define the microstructure of an alternating E-*co*-N copolymer containing *meso* (*m*) and *racemic* (r*)* alternating sequences [26]. 

**Scheme 5:**
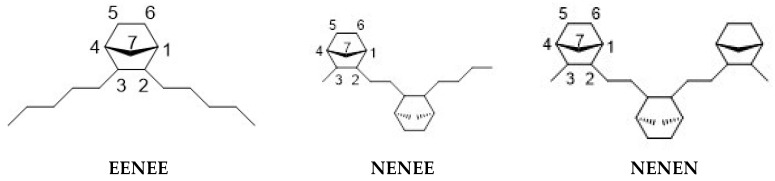
Examples of segments of E-*co*-N copolymer chain at pentad level.

**Figure 3:**
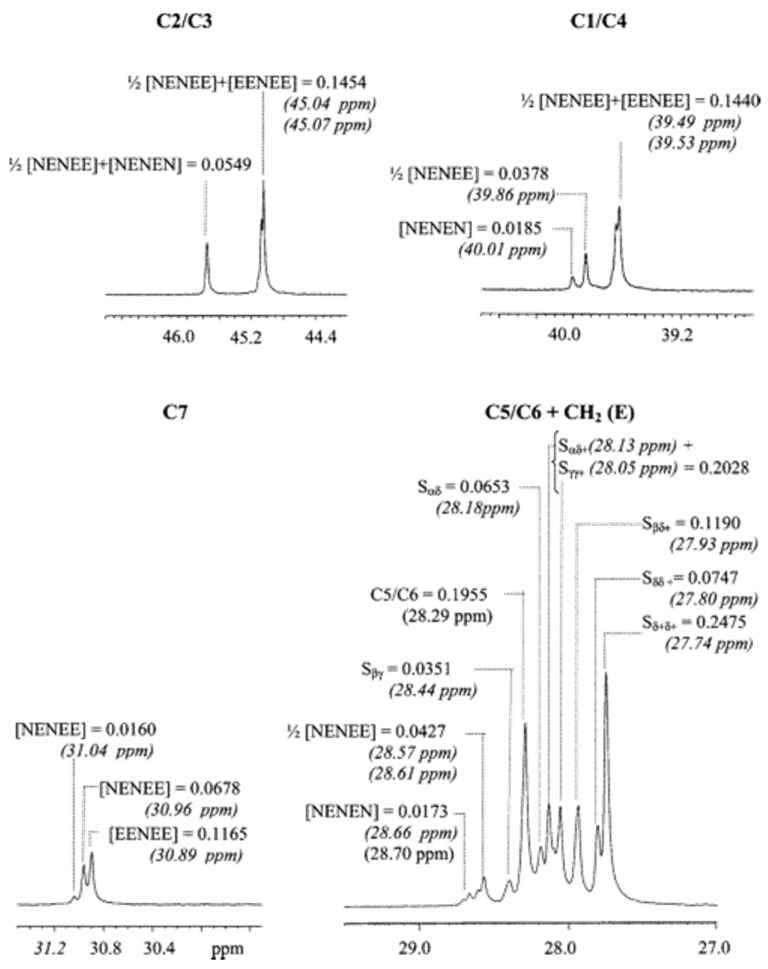
Quantitative analysis of the spectrum of a sample of an E-*co*-N copolymer prepared with **4** (N = 20.4 mol % [34]).

Catalytic systems composed of *C*_1_ symmetric *i*-Pr[(3-R-Cp)(Flu)]ZrCl_2_ (with R = Me (**4**) or *i*-Pr (**5**)) and methylaluminoxane are able to produce *isotactic alternating* copolymers with norbornene incorporation up to 40 mol % (Scheme 5). In Figure 3, the quantitative analysis at *pentad level* of the spectrum of a sample of an E-*co*-N copolymer prepared with metallocene **4** with 20.4 mol % of norbornene content is reported.

This analysis was achieved by writing for each peak area one linear equation, which relates the NPA to the variables chosen to describe the microstructure in terms of sequence distribution. Least-squares fitting of the set of equations so obtained provided the best solution for the molar fraction of each sequence. In the absence of NN diads, only six variables are needed to represent quantitatively the areas of the signals observed in the spectra of the copolymers investigated. The variables chosen were: 

*f*(m) = (NEN) = (ENENE); 

*f*0 = (NEEN) = 1/2(NEENE); 

*f*1 = (NEEEN); 

*f*(isl) = 1/2(NEE) = 1/2(ENEE); 

*f*E(isl) = the total amount of isolated E; 

*f*N(isot) = (NENEN).

The full exploitation of information contained in the E-*co*-N copolymer ^13^C NMR spectra allowed us to make assignments at pentad level (see Figure 3). Such a level of assignments allowed us to select the best statistical model describing E-*co*-N copolymerization with C_1_ symmetric catalysts, to study the influence of ligand substituents on the polymerization mechanism and to establish the importance of *chain migration mechanism versus chain retention mechanism* [32].

#### 2.2.2. Microstructure at Tetrad Level of a Random E-*co*-N Copolymers with Mid N Content

**Figure 4:**
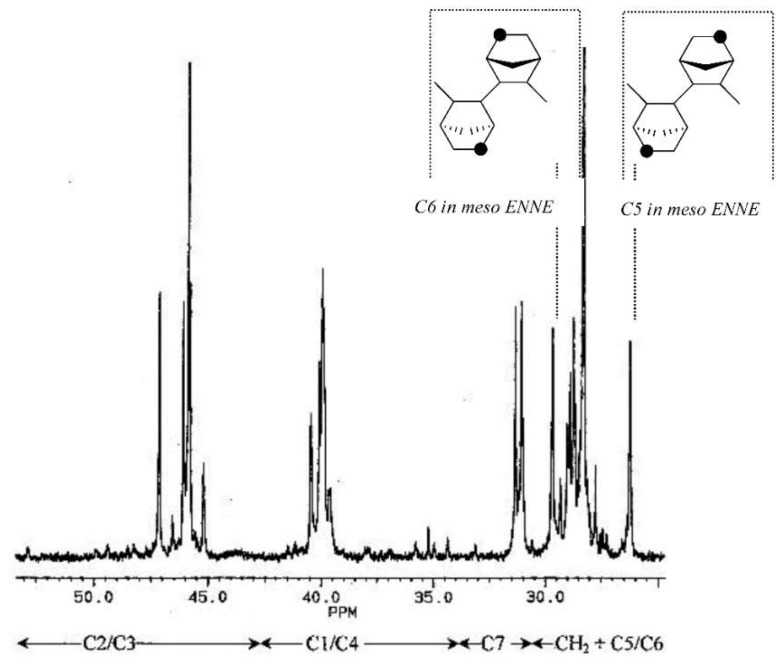
^13^C NMR spectrum of a poly(E-*co*-N) with 50.8 mol % of N produced by *rac*-Et(Indenyl)_2_ZrCl_2_ (**1**) [34].

In Figure 4, the ^13^C NMR spectrum of a poly(E-*co*-N) with 50.8 mol % of norbornene produced by catalyst *rac*-Et(Indenyl)_2_ZrCl_2_ (**1**) is shown.

**Figure 5:**
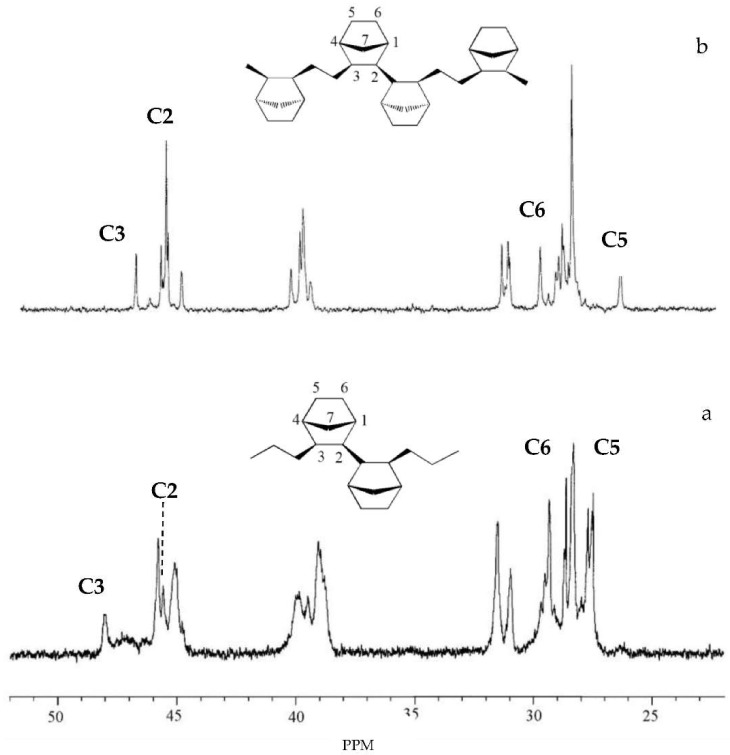
^13^C NMR spectra of a poly(E-*co*-N) produced by catalyst *i*-Pr[(Cp)(Flu)]ZrCl_2_ (**3**) with 58 mol % of N) (**a**); and by catalyst *rac*-Et(Indenyl)_2_ZrCl_2_ (**1**) with 33 mol % of N (**b**) [34].

Progress in chemical shift assignments of these copolymer spectra has included the discrimination of *meso/racemic* relationships between norbornene units in alternating NEN and in ENNE sequences. In Figure 5, the ^13^C NMR spectra of a poly(E-*co*-N) with 58 mol % of norbornene produced by catalyst *i*-Pr[(Cp)(Flu)]ZrCl_2_ (**3**) (a) and a poly(E-*co*-N) with 33 mol % of norbornene produced by catalyst *rac*-Et(Indenyl)_2_ZrCl_2_ (**1**) (b) are shown [25].

#### 2.2.3. Microstructure at Tetrad Level of a Random E-*co*-N Copolymers with High N Content

**Figure 6:**
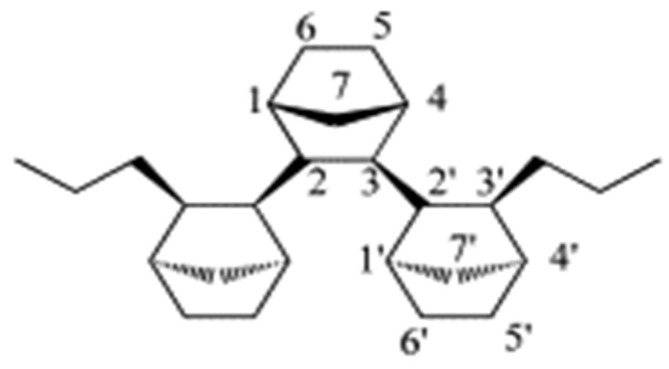

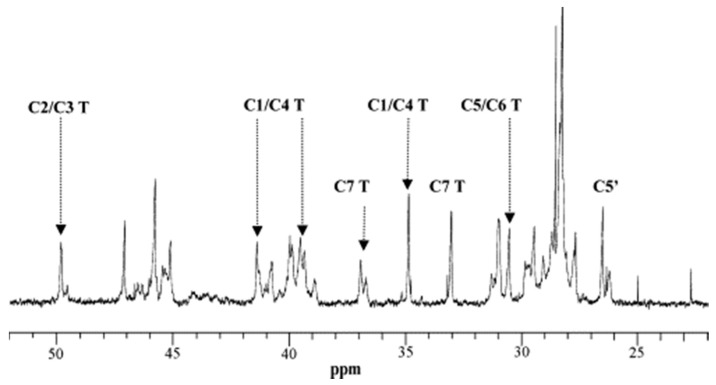
^13^C NMR spectrum of an E-*co*-N copolymer prepared with **2** containing 40.2 mol % of N [34].

The microstructure of a series of E-*co*-N copolymers synthesized in the presence of *rac*-Me_2_Si(2-Me-[e]-Indenyl)_2_ZrCl_2_ (**2**) was dominated by the high amount of meso-meso NNN sequences as is visible in the spectrum of Figure 6. The increase of possible stereosequences as the norbornene block length increases and the presence of internal and external norbornene units cause a large number of signals. Some assignments of signals of norbornene triads are listed in Table 1.

### 2.3. Microstructural Analysis of P-co-N Copolymers

**Scheme 6:**
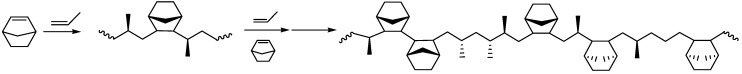
A typical random P-*co*-N copolymer chain.

The insertion of norbornene into the isotactic polypropylene chain was expected to give P-*co*-N copolymers with *T*_g_ values higher than those of E-*co*-N copolymers with the same N content and molar mass since polypropylene has *T*_g_ higher than polyethylene. However, differences in stereo- and regioregularity of propylene units as well as in the comonomer distribution and the stereoregularity of norbornene, result in complex microstructures of the polymer chain and spectra even more complex than those of E-*co*-N copolymers (Scheme 6). Thus, a detailed interpretation of P-*co*-N copolymer spectra was more difficult to achieve. For simplicity characteristics of P-*co*-N copolymers synthesized with *ansa*-metallocenes of *C*_2_ symmetry, proven effective for producing prevailingly isotactic and regioregular polypropylene are discussed.

#### 2.3.1. P-*co*-N Copolymers with Isolated N Units

**Scheme 7:**
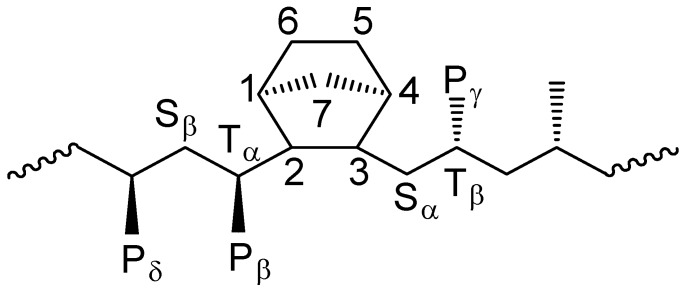
Schematic representation of a regular P-*co*-N copolymer chain along with the used numbering of carbons.

In Scheme 7, a schematic representation of a regular P-*co*-N copolymer chain (*PPNPP*) along with the numbering of carbon atoms used is shown. Norbornene has been inserted *cis*-2,3-*exo* into the metal-carbon bond. The methyls of the two propylene consecutive monomer units are in erythro as in an isotactic polypropylene chain as well as in erythro relationship with the norbornene unit.

**Figure 7:**
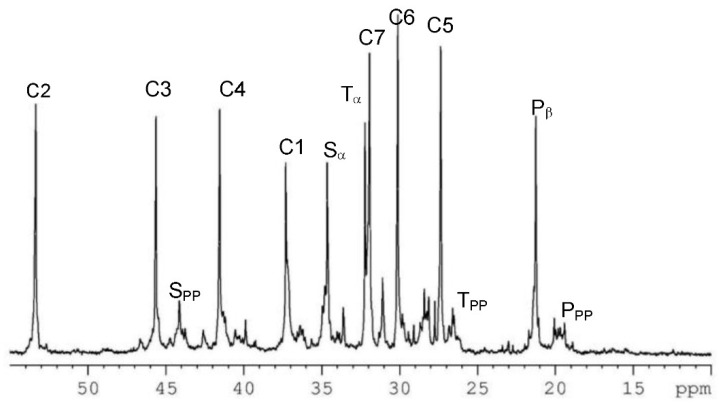
The ^13^C NMR spectrum of a P-*co*-N copolymer obtained by **1** containing 41 mol % of N [34].

Figure 7 displays the ^13^C NMR spectrum of a P-*co*-N copolymer prepared with **1**, containing about 40 mol % of N, along with the final signal assignment. 

#### 2.3.2. Microstructure at Triad Level P-*co*-N Copolymers with Mid-Low N Content Synthesized with C_2_ Symmetric Metallocenes

**Scheme 8:**
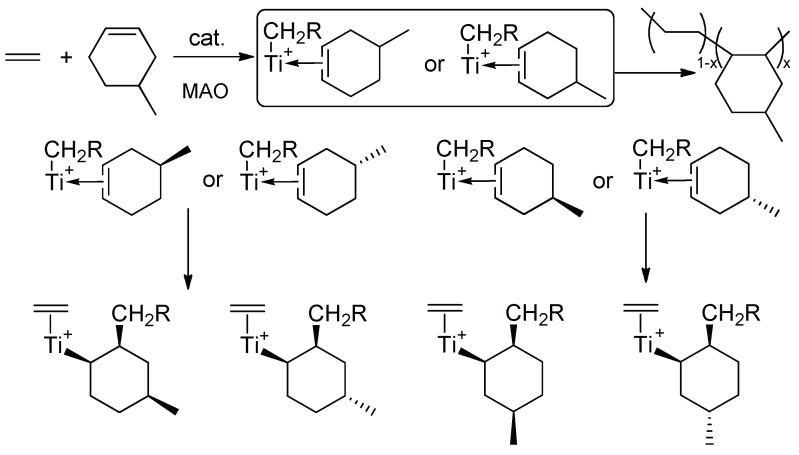
Possible *cis* (1,2- and 2,1-) insertion modes in copolymerization of ethylene with 4-methylcyclohexene (4-MeCHE) [34].

The spectrum in Figure 7 shows seven groups of signals with similar areas due to carbons of norbornene inserted as in the structure depicted in Scheme 8. DEPT experiments and comparison of the chemical shifts of these signals with those of E-*co*-N copolymers allowed us to assign the main signals of these copolymers. Then, ab initio theoretical ^13^C NMR chemical shifts, combined with R.I.S. statistics of the P-*co*-N polymer chain, gave detailed indications for the final ^13^C NMR assignment spectra of copolymers with N isolated units [35,36,37]. In detail, C6 and C5 methylenes resonate at 30.10 and 27.34 ppm, respectively, while the C7 methylene appears at 31.91 ppm. The C1 and C4 methynes resonate at 37.17 and 41.32 ppm, respectively, while C3 and C2 methynes appear at 45.40 and 53.32 ppm, respectively. In addition to signals of polypropylene, the methyl *P*_β_ carbon atom appears at 21.24 ppm. 

An apparently great difference between the values of comonomer content obtained from the areas of norbornene signals and those calculated from the propylene methyl signals suggested the existence of propylene 1,3 misinsertions in the Mt-N bond. 

A general scheme, based on the above assignments and on the peak area measurements of their ^13^C NMR spectra, was set to describe the microstructure at triad level of P-*co*-N copolymers with mid-low N content, synthesized with *C*_2_ symmetric metallocenes and thus giving mainly isospecific P homosequences. The scheme, based on the above assignments and the peak area measurements of their ^13^C NMR spectra, includes: (i) definition of the possible triads composing the copolymer chain; (ii) use of NMR techniques to assign new signals; and (iii) a best-fitting procedure to determine the copolymer microstructure [38].

**Chart 1:**
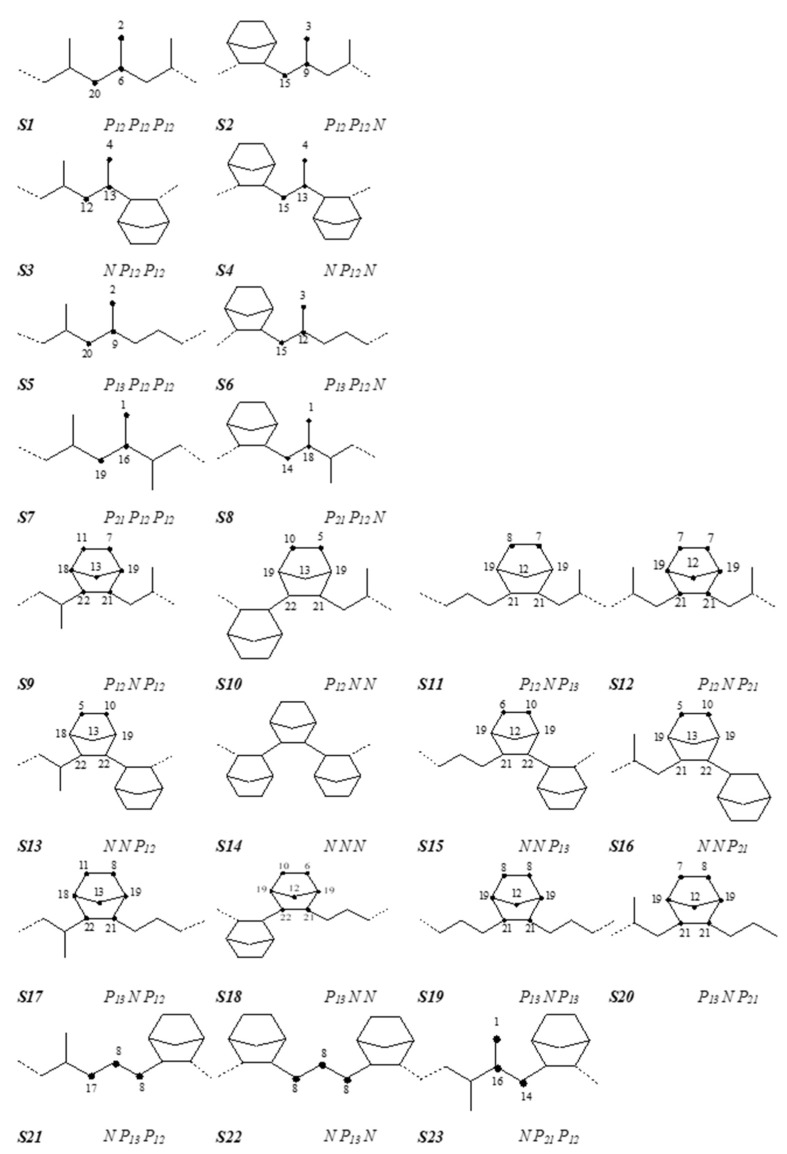
Possible triads of a random P-*co*-N copolymer chain.

*Triad definitions*: The P-*co*-N copolymer chain sketched in Scheme 7 has a typical random copolymer sequence distribution that we have described at triad level (Chart 1), initially ignoring differences in tacticity.

Since propylene *P* may be inserted in the copolymer chain 1,2 (P_12_*)*, 1,3 (P_13_*)*, and 2,1 (P_21_*),* a copolymer chain with four monomer units (*M*) P_12_, N, P_13_, and P_21_ is possible. On the basis of previous works on P-*co*-N copolymerization [36], it was assumed that units P_13_ and P_21_ may be inserted only after *N* and that N can be inserted only after P_12_. Therefore, only nine diads (P_12_P_12,_ P_12_N_,_ NP_12_, NN, NP_13_, NP_21_, P_13_P_12_, P_13_N and P_21_P_12_) and 23 triads, depicted in Chart 1, are possible. In addition, because of the asymmetry of the bonds between N and P_12_ (or P_21_), in general two diads *M*_1_*M*_2_ and *M*_2_*M*_1_ are not equivalent. In the chart, each triad *M_1_M_2_M_3_* is represented as *M*_3_*M*_2_*M*_1_, i.e., running from right to left (the catalyst metal being bound to the left side of the chain).

For each copolymer sample, ^13^C NMR signals were associated with the chemical environment of the carbons originating the signals, given in Chart 1, and used to generate a set of equations whose best-fitting solution determines the microstructure of the copolymer chain:

$$\mathrm{For}\;\mathrm{example},\;NPA\left({\mathrm{CH}}_{3}\right)=\frac{\left[f\left(P_{12}\right)+f\left(P_{21}\right)\right]}{4f\left(N\right)+3}$$

On the basis of the assignments available, each atom of the central monomer *M*_2_ was given a code number representing the signal (or group of signals) assigned to that atom in the environment of triad *M*_1_*M*_2_*M*_3_ (Chart 1). Since assignments were far from being at the complete triad level, the same code could involve two or more triads. Two-dimensional NMR techniques, including homonuclear ^1^H-^1^H and heteronuclear ^1^H-^13^C experiments have also been helpful in extending assignments [39,40].

**Figure 8:**
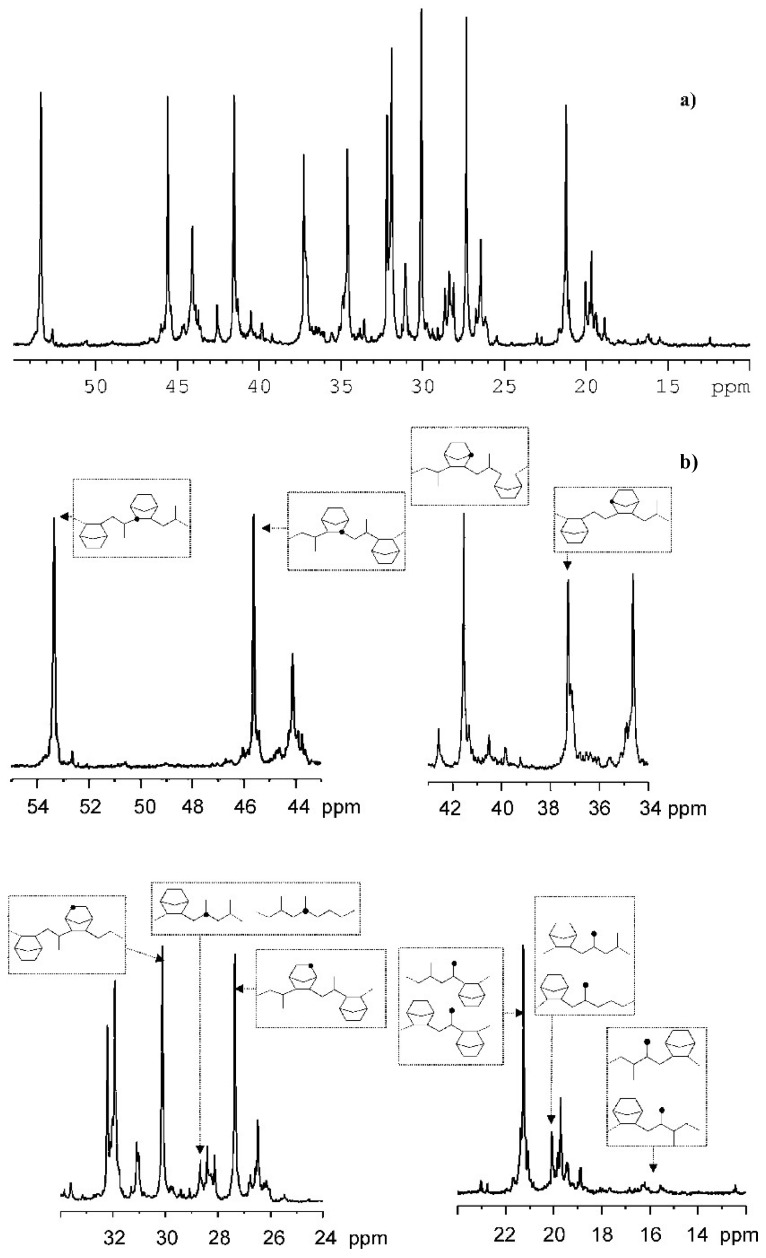
Complete (**a**) and expanded regions (**b)** of a ^13^C spectrum of a P-*co*-N copolymer prepared with **1** and containing 34% of N [34].

The main novel assignments obtained and shown in Figure 8 are summarized below:

(i) the resonances at 16.22 and 16.34 ppm due to the methyl carbon atom of central P in P_21_P_12_N (S8), and NP_21_P_12_ (S23), respectively;
(ii) the signal at 20.05 ppm due to the methyl carbon atom (Pγ) of the P in P_12_P_12_N (S2);
(iii) the signal at 21.26 ppm of the Pβγ of alternating triad NP_12_N (S4);
(iv) the signals from 21.05 to 21.94 ppm to P_β_ methyls in triad NP_12_P_12_ (S3) adjacent to a variable number of P_12_ units all in isotactic relationship;
(v) the signal at 20.25 ppm to the methyl of triad NP_12_P_12_ (S3) adjacent to a variable number of P_12_ units having different tacticity;
(vi) (the signal at 25.45 ppm to the C5 of central N in P_12_NN (S10);
(vii) the signal centered at 32.21 ppm to the CH of central P in NP_12_N (S4);
(viii) the signals at 33.60 and 33.90 ppm to CH_2_ of P in the triads NP_21_P_12_ (S23) and P_21_P_12_N (S8), respectively;
(ix) the signal at 35.70 ppm to the Sαγ methylene of a 1,3 propylene inserted units in NP_13_P_12_ triad (S21).

### 2.4. Microstructural Analysis of Poly(Ethylene-co-4-MechCHE) and Poly(Ethylene-co-MeCPE) 

Recently, Nomura developed catalysts that enable incorporations (especially with 1,2- or 2,1-insertion) of di-, tri-substituted olefins, traditionally inactive monomers in transition metal catalyzed coordination polymerization. [41,42,43,44,45] In particular, 4-methylcyclohexene (4-MeCHE) and 1-methylcyclopentene (1-MeCPE) copolymers have been synthesized recently with nonbridged half-titanocenes containing anionic donor ligands of type, Cp’TiX2(Y) (Cp’ = cyclopentadienyl group, Y = aryloxo, ketimide, phosphinimide etc.) [46]. Here, elucidation of microstructure of poly(E-*co*-4-MeCHE)s and poly(E-*co*-1-MeCPE)s is reported.

**Chart 2:**
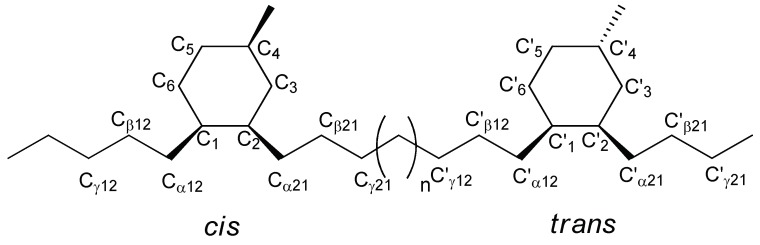
A poly(E-*co*-4-MeCHE) chain, with isolated comonomer incorporation, showing two possible stereoisomers.

**Figure 9:**
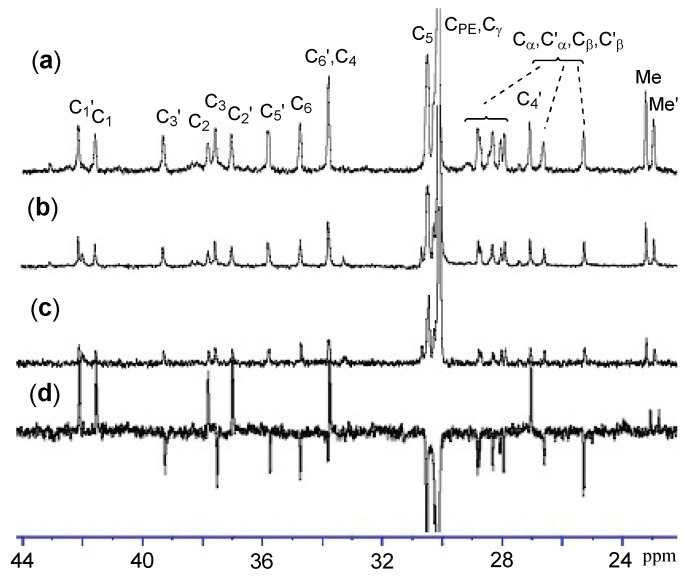
^13^C NMR spectra (**a**–**c**) and (**d**) the DEPT spectrum for poly(E-*co*-4-MeCHE)s prepared by (**8**) [34].

Figure 9 shows the ^13^C NMR spectra for poly(ethylene-*co*-4-methylcyclohexene) (poly(E-*co*-4-MeCHE) with different comonomer contents prepared with (*^t^*BuC_5_H_4_)TiCl_2_(O-2,6-Cl_2_C_6_H_3_) (**8**)-MAO catalyst system, and a DEPT spectrum [46]. In Chart 2, a poly(E-*co*-4-MeCHE) chain, with isolated comonomer incorporation, showing two possible stereoisomers, is displayed.

In order to assign the spectra of Figure 9, it was necessary to consider that, after one or more 4-MeCHE insertions, assumed to be *cis*, the methyl in position 4 of CHE may originate different stereoisomers. Indeed, as shown in Scheme 8, there are four possible types of insertion for 4-MeCHE: *cis*-1,2 or *cis*-2,1 insertions with methyl position *cis*/*trans* to Ti-alkyls.

In copolymers with a low content of 4-MeCHE, as those synthesized, the four possible 4-MeCHE additions will give rise to two possible stereoisomers of the sequence EE(4-MeCHE)EE, that we call *cis* or *trans* depending on whether Me in 4-position is *cis* or *trans* to the first CH_2_ of the polymer chain (see Chart 2). Such a difference can greatly affect the carbon chemical shifts.

The two chair forms, the most stable conformations of the cyclohexane ring of each stereoisomer, have been considered in a qualitative way. The stability of the conformer and thus the average properties of each isomer depend on the axial or equatorial positions assumed by the Me, CH_2(12)_, and CH_2(21)_ substituents and on their interactions (named C_α21_ and C_α12_ in Chart 2). The methyl in position 4 causes steric interactions when in the axial position. When the methyl of the *cis* isomer is axial with the axial CH_2_, this conformer can be ignored. Vice versa, when the methyl of the *trans* isomer is axial, it only shows the less-stable gauche interactions with the ring, which cannot be omitted.

Taking into account these conformational considerations, the two methyls at 22.69 and 23.01 ppm observed in the ^13^C spectrum of poly(E-*co*-4-MeCHE) in Figure 9, were assigned to *trans* and *cis* stereoisomers, respectively. 2D data allowed us to assign the other signals: (i) from the correlations of the two methyls in the HMBC spectrum, along the proton dimension, it was possible to assign the closest carbon atoms C_3_, C_4_, and C_5_; (ii) the methyl protons at 0.82 ppm, (CH_3_ at 22.69 ppm) correlate with three carbons at 26.77, 35.65, and 39.34 ppm, respectively, and allowed us to assign the C_4_ atom, C_5_ and C_3_ of *trans* stereoisomer; (iii) from HMBC spectrum, the resonances of the *cis* stereoisomer positioning at 30.36, 33.59, and 37.56 ppm were assigned to C_5_, C_4_, and C_3_, respectively; (iv) the other signals were assigned as follows: 42.18 ppm to C_1_; 37.14 ppm to C_2_; 33.73 ppm to C_6_ of the *trans* stereoisomer; 41.58 ppm to C_1_; 37.89 ppm to C_2_; 34.64 ppm to C_6_ of the *cis* stereoisomer. 

**Chart 3:**
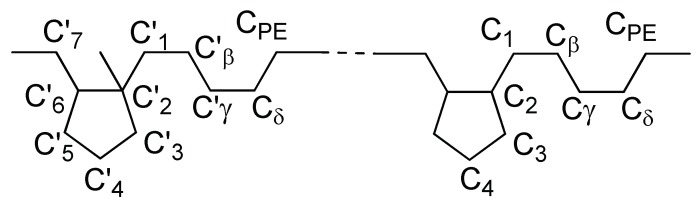
A poly(ethylene-*co*-1-MeCPE) chain, showing two possible MeCPE incorporations.

**Figure 10:**
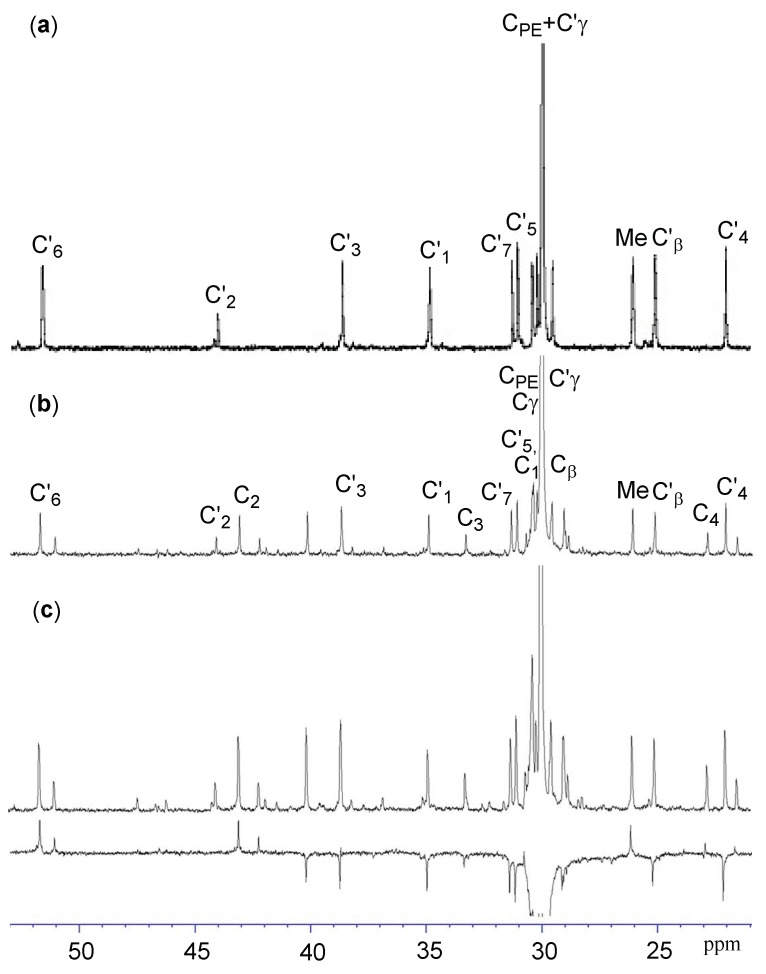
^13^C NMR spectra and the dept spectrum for poly(ethylene-*co*-1-MeCPE)s prepared by: (**a**) (*^t^*BuC_5_H_4_)TiCl_2_(*O*-2,6-Cl_2_C_6_H_3_)-MAO catalyst system (**8**), (**b**,**c**) (1,2,4-Me_3_C_5_H_2_)TiCl_2_(O-2,6-Cl_2_C_6_H_3_) (**7**)-MAO catalyst system [34].

Figure 10 shows ^13^C NMR spectra and a DEPT for poly(ethylene-*co*-1-MeCPE) prepared by (*^t^*BuC_5_H_4_)TiCl_2_(O-2,6-Cl_2_C_6_H_3_)-MAO (**8**) and (1,2,4-Me_3_C_5_H_2_)TiCl_2_(O-2,6-Cl_2_C_6_H_3_) (**7**)-MAO catalyst systems. Chart 3 describes possible insertion patterns in incorporation of 1-MeCPE in the copolymerization.

Most resonances were assigned on the basis of DEPT spectra and comparison with those of poly(ethylene-*co*-CPE) [47,48,49,50,51,52,53]. 

The spectrum of the copolymer prepared with (**8**)–MAO catalyst system in Figure 10a is rather simple. There are nine peaks in addition to the strong (CH_2_)_n_ peak at about 30 ppm of PE, arising from the different environment of *cis* 1,2 insertion of 1-MeCPE. The methyl on CPE resonates at 25.7 ppm, while from DEPT spectrum and by comparison with spectra of poly(E-*co*-CPE), resonances at 51.42 and 31.3 ppm are easily assigned to C_6′_ and C_7′_, respectively. From 2D NMR spectroscopy and from additive rules, by adding the methyl effect to the chemical shifts of a poly(ethylene-*co*-CPE) taken as a model, it was possible to assign the other carbon atoms. From the HMBC spectrum and the long-term correlations of the methyl protons, it was possible to assign C_2′_ at 43.77 ppm; C_3′_ at 38.17 ppm; and C_1′_ at 34.18 ppm.

The other signals are easily assigned for comparison to spectra of poly(ethylene-*co*-CPE). The spectra of copolymer prepared with **7**- MAO catalyst system (Figure 10b,c) show resonances assignable to 1,2- and 1,3-incorporations (see Chart 3). The spectra of copolymer prepared by **8**-MAO catalyst system show signals of only 1,2- (or 2,1-) insertion.

### 2.5. Microstructural Analysis of Poly(E-ter-N-ter-HED)

**Scheme 9:**
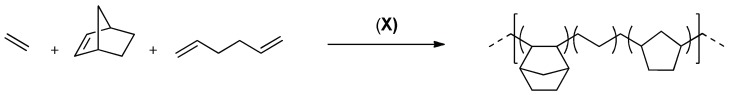
Ethylene/Norbornene/1,5-hexadiene terpolymerization performed by the *ansa*-metallocene precursor (**X**) activated by MAO.

**Scheme 10:**
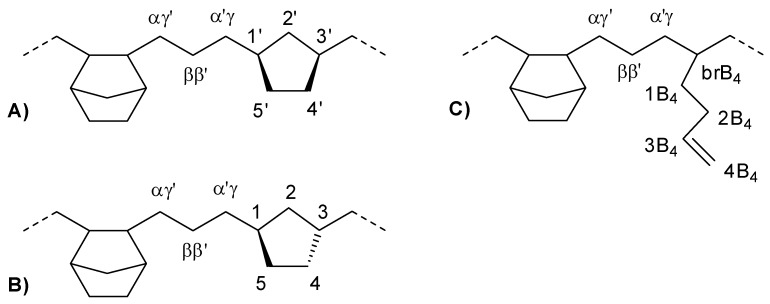
General molecular structures obtained in poly(E-*ter*-N-*ter*-HED) and relative assignment of carbon atoms. (**A**) *cis*-1,3-cyclopentane unit; (**B**) *trans*-1,3-cyclopentane unit; (**C**) 1-butenyl branch.

In this last section, attempts to determine the microstructure of ethylene/norbornene/1,5-hexadiene terpolymers poly(E-*ter-*N-*ter*-HED) synthesized by an *ansa* metallocene precursor (**X**) activated by MAO are shown [51]. HED can be inserted as a linear α-olefin; however, after insertion, cyclization can occur. In this case, it is necessary to verify the selectivity for the formation of *cis* or *trans* cyclopentane structures into the polymer chain (Scheme 9 and Scheme 10).

In order to assign the ^13^C NMR spectra of poly(E-*ter-*N-*ter*-HED), terpolymers with different norbornene content were synthesized. The poly(E-*ter-*N-*ter*-HED) spectra were compared to those of poly(HED), and of poly(N-*co*-HED), synthesized with the same catalyst.

**Figure 11:**
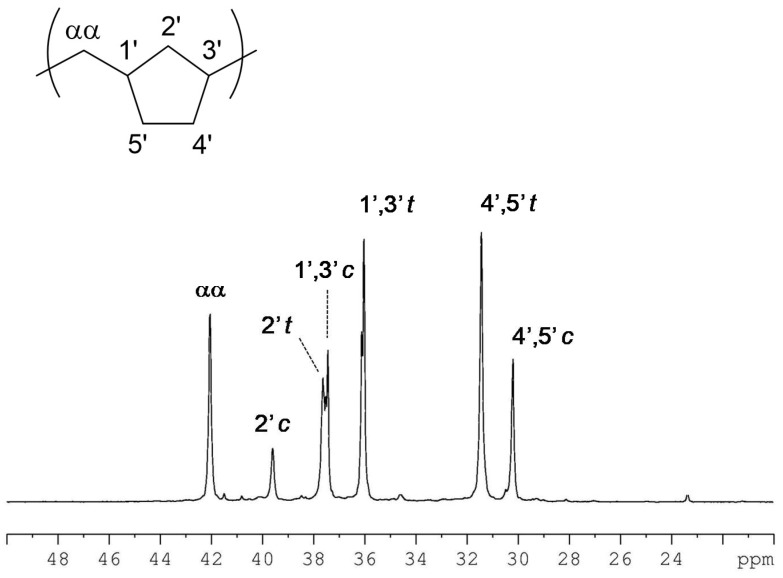
^13^C NMR spectrum (108.58 MHz, C_2_D_2_Cl_4_, 103 °C) of poly(HED) sample prepared by **5/**MAO catalytic system.

The ^13^C NMR spectrum of poly(HED) depicted in Figure 11, assigned for comparison to literature data [52,53,54], proved clearly that HED was mainly incorporated as 1,3-cyclopentane (CP) units. This means that intramolecular cyclization of 1,2-inserted HED occurred before the next monomer insertion, and only a small fraction of the incorporated diene formed vinyl terminated branches (Vy) along the polymer backbone. The cyclopentane structures were assigned as follows: *cis* rings, 30.19 ppm (4′,5′-*c*), 37.43 ppm (1′,3′-*c*), 39.5 ppm (2′-*c*); *trans* rings, 31.43 ppm (4′,5′-*t*), 36.02 ppm (1′,3′-*t*), 37.62 ppm (2′-*t*); methylene bridge carbon, 42.02 ppm (αα). Polymers were characterized by a predominance of *trans* rings, determined by diastereoselectivity of the intramolecular cyclization step.

**Figure 12:**
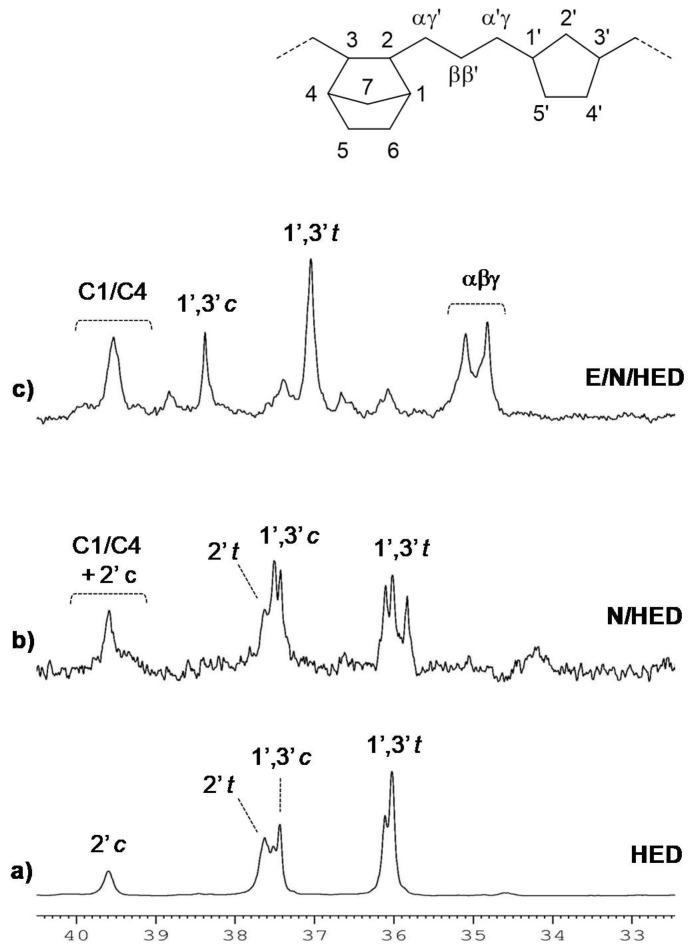
Expansions of the region between 32 and 41 ppm of ^13^C NMR spectra (108.58 MHz, C_2_D_2_Cl_4_, 103 °C) of polymers prepared by **X/**MAO: (**a**) poly(HED); (**b**) poly(N-*co*-HED); (**c**) poly(E-*ter-*N-*ter*-HED).

In Figure 12, the expansion in the region between 32 and 41 ppm of the ^13^C NMR spectra of poly(HED) (a); poly(N-*co*-HED) (b); and of poly(E-*ter-*N-*ter*-HED) (c) prepared under similar polymerization conditions are compared.

The spectra clearly demonstrate that resonance positions of signals ascribed to 1,3-cyclopentane units of HED are highly sensitive to compositional and stereochemical effects. It appears from Figure 12 that the NMR frequencies of carbon atoms 1′,3′-*cis* and 1′,3′-*trans* of poly(N-*co*-HED) depicted in (b) are slightly shifted upfield by c.a. 0.05 ppm compared to those of poly(HED) depicted in (a). This effect is certainly related to the placements of norbornene units close to C_1_ and C_3_ positions of the cyclopentane ring. On the other hand, resonances of carbon atoms 1′,3′-*cis* and 1′,3′-*trans* are shifted downfield by over 1 ppm in the case of poly(E-*ter-*N-*ter*-HED), depicted in Figure 12c. This large effect is due to the placements of different substituents at C_1_ and C_3_ positions of the cyclopentane ring, which are likely ethylene units in the present situation. In this regard, signals appearing between 34.8 and 35 ppm, indicated in Figure 12c with αβγ, were ascribed to methylene carbons from the main chain immediately adjacent to cyclopentane units, and in proximity to norbornene. Interestingly, in the case of poly(E-*ter-*N-*ter*-HED) in Figure 12c, the presence of several weak signals in the region between 36 and 39 ppm is most likely associated with the shifting of the resonance of cyclopentane units depending on the microstructure of the terpolymer. 

**Scheme 11:**
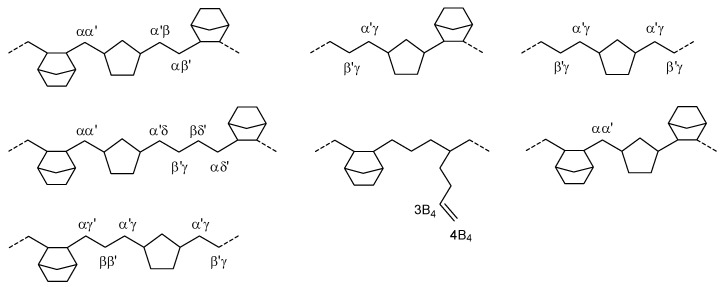
Possible chain fragments of a poly(E-*ter-*N-*ter*-HED) chain containing low amount of 1,5-hexadiene incorporated.

However, it is difficult to assign each carbon signal arising from the 1,3-cyclopentane units. The peak intensities are often weak because of the relatively low level of CP units in the terpolymers, and several signals are completely hidden by adjacent resonances of norbornene units. The various types of possible chain fragments that may form in the terpolymer are summarized in Scheme 11.

**Figure 13:**
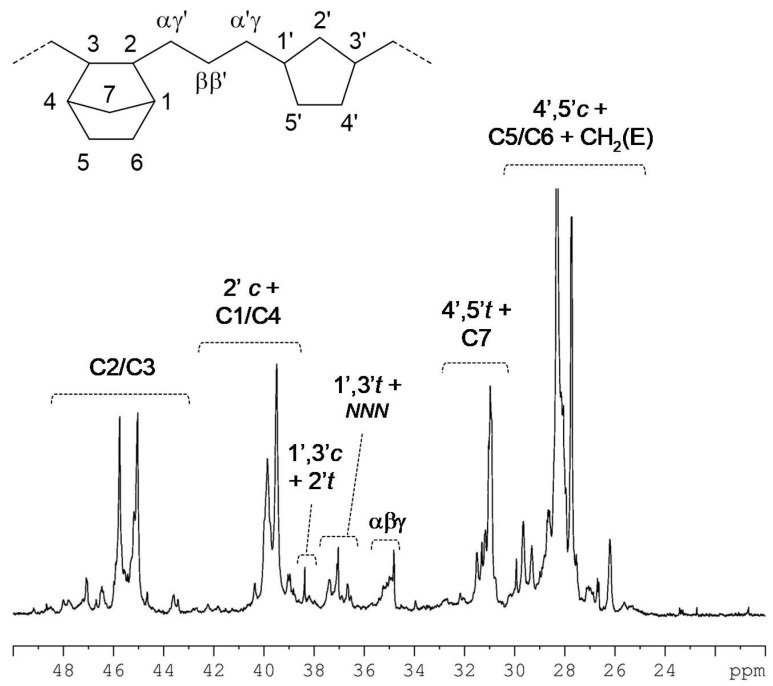
^13^C NMR spectrum (108.58 MHz, C_2_D_2_Cl_4_, 103 °C) of poly(E-*ter-*N-*ter*-HED) sample prepared by **X/**MAO catalytic system at N/E/HED = 4/1/4.

The whole ^13^C NMR spectrum of a sample, prepared by **X/**MAO catalytic system at N/E/HED = 4/1/4, is shown in Figure 13 with the general assignment for each of the resolved groups of peaks. The signal that appeared at 38.36 ppm was identified as the methyne carbons 1′,3′-*cis* of the cyclopentane unit. The adjacent weak signal at 38.18 ppm should correspond to the methylene carbon 2′-*trans*, while the 2′-*cis* carbon signal was probably located around 39.3 ppm, hidden by the resonances ascribed to the C1/C4 norbornene carbon atoms.

The signal assigned to carbons 1′,3′-*trans* appearing at 37.03 ppm was located in the cluster of peaks between 36.5 and 37.4 ppm, relative to C1/C4 and C7 norbornene *NNN* triads. A weaker signal around 36.5 ppm was also noted. In this region, a separate integration of peaks was not feasible; thus, the intensity of the resonance associated with the 1′,3′-*trans* carbon atoms was impossible to estimate. The methylene resonance of 4′,5′-*trans* carbon atoms was expected to fall in the range between 31.5 and 30.5 ppm, within the same interval of the C7 carbon resonances of norbornene, while the resonance of carbons 4′,5′-*cis* was expected to fall around 29.8 ppm, completely obscured by the E-N backbone resonances. A new group of signals, indicated with symbol αβγ, was seen in the range between 34.81 and 35.4 ppm, which are probably related to carbons from the main chain (α’β, αβ’, α’γ, α’δ, ββ’, and β’γ) immediately adjacent to cyclopentane and in proximity to the norbornene unit; in the same interval, a weak signal arising from C1/C4 *NNN* triads was expected. Resonances assignable to pendant double bonds (Vy), which are due to HED polymerization without cyclization, were assigned to unsaturated carbon atoms appearing in the region between 110 and 150 ppm. Thus, signals at 112.1 and 137.6 ppm were attributed to the side chain double bond carbon atoms 4B_4_ and 3B_4_ sketched in Scheme 10. These assignments allow us to conclude that 1,5-hexadiene can be incorporated into the polymer chain as 1-butenyl branches (Vy) or in the form of cyclopentane structures (CP) connected at C_1_ and C_3_ positions to the polymer backbone and to calculate compositions of poly(E-*ter*-N-*ter*-HED) on the basis of the peak integrals of carbons in the ^13^C NMR spectra.

## 3. Conclusions

Despite the unique versatility of ^13^C NMR spectroscopy whenever a new polymer structure is synthesized, it is challenging to assign the specific polymer microstructure from ^13^C NMR experiments. Here, an overview is given of the efforts in elucidating the microstructure of cyclic olefin based copolymers through ^13^C NMR analysis. Their spectra are quite complex due to the presence in the polymer chain of stereogenic carbons per monomer unit and because the chemical shifts of these copolymers do not obey simple additive rules. The examples presented illustrate how it is possible to assign ^13^C NMR spectra of these copolymers through a methodology, which includes synthesis of copolymers with different comonomer content and by catalysts with different symmetries, the use of mono- or bidimensional NMR techniques, the consideration of conformational characteristics of the copolymer chain, the exploitation of all the peak areas of the spectra by accounting for the stoichoimetric requirements of the copolymer chain and the best fitting of a set of linear equations obtained. In particular, the focus is on the elucidation of E-*co*-N and P-*co*-N copolymer microstructures through ^13^C NMR analysis. Advances in signal assignments of the complex spectra of these copolymers are reviewed along with the various methodologies utilized to achieve them. In addition, the methods used for understanding the microstructures of poly(ethylene-*co*-4-MeCHE)s, poly(ethylene-*co*-1-MeCPE)s, and of poly(E-*ter*-N-*ter*-HED) from ^13^C NMR are reported. The microstructural description of the copolymer chain, attained from such assignments, is important for acquiring information on the copolymerization mechanisms and on understanding relationship between polymer microstructures and polymer properties.

## Figures and Tables

**Table 1:** Assignments of ^13^C NMR of spectra of random E-*co*-N copolymers with high N content.

Carbon	Chemical shift	Carbon	Chemical shift	Carbon	Chemical shift	Carbon	Chemical shift
C5′ *m,m*	26.59	C7 *m,m*	33.07	C1/C4 *m,m*	34.92	C2/C3 T	45.42/47.13
C5′ *r,m*	27.20–27.70	C7 T	34.34	C1/C4 T	35.18	C2/C3 T	49.34
C5/C6 T	29.37	C7 T	35.70	C1/C4 T	36.94	C2/C3 *m,m*	49.80
C5/C6 T	29.49	C7 *m,m*	36.74	C1/C4 T	37.87	C2/C3 T	50.00
C5/C6 T	30.50	C7 *m,m*	36.94	C1/C4 T	39.28–39.39	C2/C3 *m,m*	52.70–52.84
C5/C6 *m,m*	30.58			C1/C4 T	40.80	C2/C3 T	53.44
				C1/C4 T	41.32		
				C1/C4 T	41.45		
				C1/C4 T	41.56		

## References

[1] Spiess H.W. (2017). 50th Anniversary Perspective: The Importance of NMR Spectroscopy to Macromolecular Science. Macromolecules.

[2] Tonelli A.E. (1989). NMR Spectroscopy and Polymer Microstructure: The Conformational Connection.

[3] Grant D.M., Paul E.G. (1964). Carbon-13 Magnetic Resonance. II. Chemical Shift Data for the Alkanes. J. Am. Chem. Soc..

[4] Lindeman L.P., Adams J.Q. (1971). Carbon-13 nuclear magnetic resonance spectrometry. Chemical shifts for the paraffins through C9. Anal. Chem..

[5] Stothers J.B. (1972). Carbon-13 NMR Spectroscopy.

[6] Bovey F.A. (1982). Chain Structure and Conformation of Macromolecules.

[7] Provasoli A., Ferro D.R. (1977). Correlation between C-13 NMR Chemical-shifts and Conformation of Polymers. 1. Methyl Spectrum of 3,5,7,9,11,13,15-Heptamethylheptadecane. Macromolecules.

[8] Tonelli A.E. (1979). Are the Steric Effects on the C-13 NMR Chemical- shifts of Hydrocarbon Polymers Really Long-range?. Macromolecules.

[9] Provasoli A., Ferro D.R., Zambelli A., Provasoli A., Locatelli P., Rigamonti E. (1980). Correlation between C-13 NMR Chemical-shifts and Conformation of Polymers. 2. Improved Method of Calculation. Macromolecules.

[10] Ferro D.R., Ragazzi M. (1984). Correlation between C-13 NMR Chemical-shifts and Conformation of Polymers. 4. Solid-state spectra of poly(3-methyl-1-Pentene). Macromolecules.

[11] Wei M., Ivey D.T., Tonelli A.E. (2002). What is the source of the microstructural dependence of resonance frequencies observed in the solution NMR of polymers whose local structures and conformations appear to be independent of their longer range microstructures?. Macromolecules.

[12] Busico V., Cipullo R. (2001). Microstructure of polypropylene. Prog. Polym. Sci..

[13] Kaminsky W., Bark A., Arndt M. (1991). New polymers by homogenous zirconocene/aluminoxane catalysts. Makromol. Chem. Macromol. Symp..

[14] Kaminsky W., Arndt-Rosenau M., Scheirs J., Kaminsky W. (2000). Homo- and copolymerization of cycloolefins by metallocene catalysts. Metallocene-Based Polyolefins.

[15] Kaminsky W., Boggioni L., Tritto I., Coates G.W., Sawamoto M. (2012). 3.26 Cycloolefin polymerization. Comprehensive Polymer Science. Volume 3. Chain Vinyl Polymerization.

[16] Boggioni L., Tritto I. (2013). State of the art of cyclic olefin polymers. MRS Bull..

[17] Tritto I., Boggioni L., Ferro D.R. (2006). Metallocene catalyzed ethene- and propene co-norbornene polymerization: Mechanisms from a detailed microstructural analysis. Coord. Chem. Rev..

[18] Wendt R.A., Fink G. (2001). C-13 NMR studies of ethene/norbornene copolymers using C-13-enriched monomers: Signal assignments of copolymers containing norbornene microblocks of up to a length of three norbornene units. Macromol. Chem. Phys..

[19] Provasoli A., Ferro D.R., Tritto I., Boggioni L. (1999). The conformational characteristics of ethylene−norbornene copolymers and their influence on the ^13^C NMR spectra. Macromolecules.

[B20-polymers-10-00647] 20.One of the reviewers pointed out that the conformational models derived by us to determine the conformational populations in the norbornene copolymers have not been independently tested and criticized the relationship employed by us between nuclear shieldings and conformations. The discussion on validity of these objections is out of the scope of this article. We emphasize that the results presented in published papers rely not only on conformational calculations but also on the overall agreement with experimental data, although probably the use of better conformational models would have led to more complete assignments.

[21] Arndt M., Gosmann M. (1999). Transition metal catalyzed polymerisation of norbornene *Polym*. Bull..

[22] Bergström C.H., Sperlich B.R., Ruotoistenmäki J., SeppäläJ V. (1998). Investigation of the microstructure of metallocene-catalyzed norbornene-ethylene copolymers using NMR spectroscopy. J. Polym. Sci. Part A.

[23] Tritto I., Boggioni L., Sacchi M.C., Locatelli P. (1998). Cyclic olefin polymerization and relationships between addition and ring opening metathesis polymerization. J. Mol. Catal. A Chem..

[24] Ruchatz D., Fink G. (1998). Ethene−Norbornene copolymerization using homogenous metallocene and half-sandwich catalysts:  Kinetics and relationships between catalyst structure and polymer structure. 2. Comparative study of different metallocene- and half-sandwich/methylaluminoxane catalysts and analysis of the copolymers by ^13^C nuclear magnetic resonance spectroscopy. Macromolecules.

[25] Tritto I., Marestin C., Boggioni L., Zetta L., Provasoli A., Ferro D.R. (2000). Ethylene−norbornene copolymer microstructure. Assessment and advances based on assignments of ^13^C NMR spectra. Macromolecules.

[26] Tritto I., Marestin C., Boggioni L., Brintzinger H.H., Ferro D.R. (2001). Stereoregular and stereoirregular alternating ethylene−norbornene copolymers. Macromolecules.

[27] Thorshaug K., Mendichi R., Boggioni L., Tritto I., Trinkle S., Friedrich C., Mülhaupt R. (2002). Poly(ethene-*co*-norbornene) obtained with a constrained geometry catalyst. A study of reaction kinetics and copolymer properties. Macromolecules.

[28] Herfert N., Montag P., Fink G. (1993). Elementary processes of the Ziegler catalysis, 7. Ethylene, α-olefin and norbornene copolymerization with the stereorigid catalyst systems Ipr[FluCp]Zrcl_2_/MAO and Me_2_Si[Ind]_2_ZrCl_2_/MAO. Makromol. Chem..

[29] Rische T., Waddon A.J., Dickinson L.C., MacKnight W.J. (1998). Microstructure and morphology of cycloolefin copolymers. Macromolecules.

[30] Ragazzi M., Carbone P., Ferro D.R. (2002). Ab initio molecular modeling of C-13 NMR chemical shifts of polymers. 1. Ethylene-norbornene copolymers. Int. J. Quantum Chem..

[31] Tritto I., Boggioni L., Jansen J.C., Thorshaug K., Sacchi M.C., Ferro D.R. (2002). Ethylene-norbornene copolymers from metallocene-based catalysts: Microstructure at tetrad level and reactivity ratios. Macromolecules.

[32] Tritto I., Boggioni L., Ferro D.R. (2004). Alternating Isotactic Ethylene−Norbornene Copolymers by C1-Symmetric Metallocenes:  Determination of the Copolymerization Parameters and Mechanistic Considerations on the Basis of Pentad Analysis. Macromolecules.

[33] Tritto I., Boggioni L., Zampa C., Ferro D.R. (2005). Ethylene-norbornene copolymers by C*s*-symmetric metallocenes: Determination of the copolymerization parameters and mechanistic considerations on the basis of tetrad analysis. Macromolecules.

[B34-polymers-10-00647] 34.The ^13^C NMR spectra were measured in C_2_D_2_Cl_4_ at 105 °C; chemical shifts are referred to HMDS.

[35] Carbone P., Ragazzi M., Tritto I., Boggioni L., Ferro D.R. (2003). Ab initio molecular modeling of ^13^C NMR chemical shifts of polymers. 2. Propene-norbornene copolymers. Macromolecules.

[36] Boggioni L., Bertini F., Zannoni G., Tritto I., Carbone P., Ragazzi M., Ferro D.R. (2003). Propene-norbornene copolymers: Synthesis and analysis of polymer structure by C-13 NMR spectroscopy and ab initio chemical shift computations. Macromolecules.

[37] Boggioni L., Tritto I., Ragazzi M., Carbone P., Ferro D.R. (2004). Propene-Norbornene Copolymers: Synthesis and Microstructure. Macromol. Symp..

[38] Boggioni L., Zampa C., Ravasio A., Ferro D.R., Tritto I. (2008). Propene-Norbornene Copolymers by C2-symmetric Metallocene *rac*-Et(Ind)_2_ZrCl_2_: Influence of Reaction Conditions on Reactivity and Copolymer Properties. Macromolecules.

[39] Boggioni L., Ravasio A., Zampa C., Ferro D.R., Tritto I. (2010). Penultimate Effects and Chain Epimerization in Propene-Norbornene Copolymers by *rac*-Me_2_Si(2-Me-Ind)_2_ZrCl_2_ C-2-Symmetric Metallocene. Macromolecules.

[40] Boggioni L., Ravasio A., Boccia A.C., Ferro D.R., Tritto I. (2010). Propene-Norbornene Copolymers. Toward a Description of Microstructure at Triad Level Based on Assignments of C-13 NMR Spectra. Macromolecules.

[41] Itagaki K., Fujiki M., Nomura K. (2007). Effect of cyclopentadienyl and anionic donor ligands on monomer reactivities in copolymerization of ethylene with 2-methyl-1-pentene by nonbridged half-titanocenes- cocatalyst systems. Macromolecules.

[42] Nomura K., Itagaki K. (2005). Efficient incorporation of vinylcylohexane in ethylene/vinylcyclohexane copolymerization catalyzed by nonbridged half-titanocenes. Macromolecules.

[43] Khan F.Z., Kakinuki K., Nomura K. (2009). Copolymerization of ethylene with *t*-buytlethylene using nonbridged half-titanocene-cocatalyst systems. Macromolecules.

[44] Kakinuki K., Fujiki M., Nomura K. (2009). Copolymerization of ethylene with α-olefins containing various substituents catalyzed by half-titanocenes: Factors affecting the monomer reactivities. Macromolecules.

[45] Nomura K., Kakinuki K., Fujiki M., Itagaki K. (2008). Direct precise functional group introduction into polyolefins: Efficient incorporation of vinyltrialkylsilanes in ethylene copolymerizations by nonbridged half-titanocenes. Macromolecules.

[46] Harakawa H., Patamma S., Boccia A.C., Boggioni L., Ferro D.R., Losio S., Nomura K., Tritto I. (2018). Ethylene Copolymerization with 4-Methyl-cyclohexene, 1-Methylcyclopentene by Half-Titanocene Catalysts: Effect of Ligands and Microstructural Analysis in the Copolymers. Macromolecules.

[47] Naga N., Imanishi Y. (2002). Copolymerization of ethylene and cyclopentene with zirconocene catalysts: Effect of ligand structure of zirconocenes. Macromol. Chem. Phys..

[48] Lavoie A.R., Waymouth R.M. (2004). Catalytic syntheses of alternating, stereoregular ethylene/cycloolefin copolymers. Tetrahedron.

[49] Fujita M., Coates G.W. (2002). Synthesis and characterization of alternating and multiblock copolymers from ethylene and cyclopentene. Macromolecules.

[50] Tang L.-M., Duan Y.-Q., Pan L., Li Y.-S. (2005). Copolymerization of ethylene and cyclopentene with bis(β-enaminoketonato) titanium complexes. J. Polym. Sci. Part A.

[B51-polymers-10-00647] 51.Catalyst **X** is considered for patent applications, so the structures of this catalyst cannot be disclosed.

[52] Naga N. (2005). Copolymerization of ethylene with cycloolefins or cyclodiolefins by a constrained-geometry catalyst. J. Polym. Sci. Part A.

[53] Liu J., Nomura K. (2007). Highly efficient ethylene/cyclopentene copolymerization with exclusive 1,2-cyclopentene incorporation by (cyclopentadienyl)-(ketimide)titanium(IV complex–MAO catalysts. Adv. Synth. Catal..

[54] Cheng H.N., Khasat N.P. (1988). C-13-NMR Characterization of poly(1,5-hexadiene). J. Appl. Polym. Sci..

